# Fosfomycin Resistance in *Escherichia coli*, Pennsylvania, USA

**DOI:** 10.3201/eid2111.150750

**Published:** 2015-11

**Authors:** Hind Alrowais, Christi L. McElheny, Caressa N. Spychala, Sangeeta Sastry, Qinglan Guo, Adeel A. Butt, Yohei Doi

**Affiliations:** University of Pittsburgh School of Medicine, Pittsburgh, Pennsylvania, USA (H. Alrowais, C.L. McElheny, C.N. Spychala, S. Sastry, Q. Guo, A.A. Butt, Y. Doi);; Fudan University, Shanghai, China (Q. Guo);; Ministry of Health, Shanghai (Q. Guo);; Hamad Healthcare Quality Institute, Doha, Qatar (A.A. Butt);; Hamad Medical Corporation, Doha (A.A. Butt)

**Keywords:** fosfomycin resistance, antimicrobial resistance, fosA3 gene, glutathione S-transferase, extended-spectrum β-lactamase, ESBL, 16S rRNA methyltransferase, Escherichia coli, bacteria, plasmids, Pennsylvania, United States

## Abstract

Fosfomycin resistance in *Escherichia coli* is rare in the United States. An extended-spectrum β-lactamase–producing *E. coli* clinical strain identified in Pennsylvania, USA, showed high-level fosfomycin resistance caused by the *fosA3* gene. The IncFII plasmid carrying this gene had a structure similar to those found in China, where fosfomycin resistance is commonly described.

Fosfomycin is a phosphonic acid derivative with antibacterial activity against a wide range of gram-negative pathogens and some gram-positive pathogens. It inhibits bacterial cell wall synthesis and is bactericidal against most *Escherichia coli* strains, as well as many strains of other members of the family *Enterobacteriaceae.* In the United States, only an oral formulation containing fosfomycin tromethamine is approved for clinical use.

Because of increasing resistance of *E. coli* strains to other commonly used agents, such as ciprofloxacin and trimethoprim/sulfamethoxazole, fosfomycin has become one of the first-line agents recommended for treatment of uncomplicated urinary tract infection. A recent study of *E. coli* strains collected at veterans’ hospitals in the United States included ciprofloxacin-resistant and extended-spectrum β-lactamase–producing strains. These strains had 98%–99% susceptibility to fosfomycin (*1*). However, susceptibility data on fosfomycin are relatively limited overall because this agent is not routinely tested in most clinical microbiology laboratories.

Several fosfomycin resistance mechanisms have been described in *E. coli*, including reduced permeability, modification of the *murA* gene target, and modification of fosfomycin (*2*). In *E. coli*, the plasmid-mediated fosfomycin resistance gene *fosA3*, which encodes a glutathione S-transferase, was first identified in a fosfomycin-resistant *E. coli* strain in Japan (*3*). This enzyme modifies fosfomycin, thus inactivating the agent and conferring high-level fosfomycin resistance. The *fosA3* gene has been reported from only countries in eastern Asia, especially China. We report a case of persistent colonization with a *fosA3*-carrying *E. coli* strain in a patient in Pennsylvania, USA.

## The Study

The patient was a woman with multiple hospitalizations related to sickle cell crises and end-stage kidney disease; she was receiving peritoneal dialysis. She produced minimal amounts of urine and had frequent urinary tract infections due to extended-spectrum β-lactamase–producing *E. coli*. She did not have history of travel to eastern Asia, from which all reports of *fosA3* have so far originated. The first available *E. coli* strain from this patient, ECRB1, was isolated from a urine sample in 2007 and was reported as a multidrug-resistant strain harboring *bla*_CTX-M-65_ and *rmtB* (*4*).

She came to a hospital in 2010 because of a power outage in her home. During this brief hospitalization, a culture collected from the peritoneal catheter exit site grew *E. coli* strain YD472. This strain was found to be highly resistant to fosfomycin, which initiated the present investigation. She had received multiple antimicrobial agents, including cefazolin, ceftriaxone, ciprofloxacin, moxifloxacin, azithromycin, and trimethoprim/sulfamethoxazole, in the prior 5 years, but she had not received fosfomycin according to available medical records.

*E. coli* YD472 was highly resistant to fosfomycin (MIC >1,024 μg/mL by Etest), which was confirmed by using the agar dilution method with Mueller-Hinton agar and glucose-6-phosphate (25 μg/mL) as an additive, as endorsed by the Clinical and Laboratory Standards Institute (*5*). Given this high-level resistance, PCR was conducted on YD472 to identify *fosA3* and *fosC2*, which have been reported as acquired fosfomycin resistance genes among recent *E. coli* strains in Japan, China, and South Korea (*3*,*6*,*7*). A PCR result was positive for *fosA3*, which was confirmed by sequencing. The *fosA3* gene was transferable to *E. coli* TOP10 by electroporation of the total plasmid extracted from YD472. The *E. coli* TOP10 transformant strain harboring the *fosA3*-encoding plasmid pYHCC was resistant to cefotaxime, gentamicin, and fosfomycin. 

We then sequenced the entire plasmid in pYHCC by using single-molecule real-time sequencing (Pacific Biosciences, Menlo Park, CA, USA) as described (*8*). Sequencing in a single cell resulted in a full-length plasmid that could then be circularized and finished (mean coverage 1,325×). The sequence was manually annotated by using RAST (http://rast.nmpdr.org/), ORF Finder (http://www.ncbi.nlm.nih.gov/gorf/gorf.html), and IS Finder (https://www-is.biotoul.fr/), and comparisons were made by using BLAST analysis (http://blast.ncbi.nlm.nih.gov/Blast.cgi). The complete plasmid sequence has been deposited in GenBank under accession no. KR078259.

**Figure 1:**
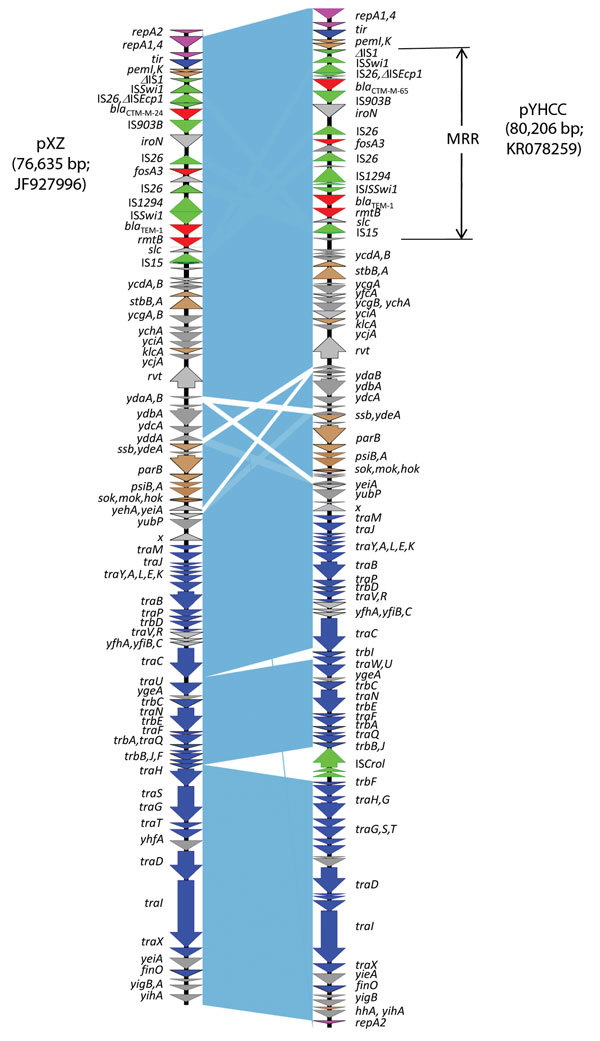
Comparative analysis of *fosA3*-carrying IncFII plasmids pYHCC with pXZ. Open reading frames are indicated by arrows and colored according to their putative functions: magenta arrows indicate genes involved in replication; blue arrows indicate genes associated with plasmid conjugal transfer. Brown arrows indicate genes involved in plasmid stability; red arrows indicate antimicrobial drug resistance genes; green arrows indicate accessory genes of mobile elements; gray arrows indicate other backbone genes and inserted foreign genes. Light blue shading indicates shared backbone regions with a high degree of homology. MRR, multidrug resistance region.

pYHCC is an 80,206-bp circular plasmid with a G + C content of 51.8% and a typical IncFII replicon, which is classified as F2:A–:B– by replicon sequence typing (*9*). It encodes 121 genes (including hypothetical genes) and harbors a 13-kb multidrug resistance region and a 67-kb R100-like backbone region. The backbone region contains genes for replication, transfer, and maintenance. This backbone of pYHCC contains 24 *tra* genes and 7 *trb* genes. The structure of pYHCC is most closely related to that of pXZ, a 77-kb IncFII, *fosA3*-carrying plasmid reported from *E. coli* strains isolated from diseased chickens and ducks in China (99% identity with 95% coverage) (Figure 1) (*10*). It also shares high similarities (99% identity with 84%–87% coverage) with other *fosA3*-carrying *E. coli* plasmids reported, including pHN7A8 from a dog in China (*11*), pHN3A11 from a cat in China, pHK23a from a pig in Hong Kong (*12*), and pFOS-HK151325 from a human in Hong Kong (*13*).

A feature that distinguishes pYHCC from pXZ and contributes to the size difference of the plasmids is insertion of IS*CroI* between *trbJ* and *trbF* in pYHCC. Plasmid pYHCC carries 4 antimicrobial resistance genes, all of which are in the multidrug resistance region. These genes are *bla*_CTX-M-65_ (conferring cephalosporin resistance), *fosA3* (conferring fosfomycin resistance), *bla*_TEM-1_ (conferring ampicillin resistance), and *rmtB* (conferring aminoglycoside resistance). The *fosA3* gene and its downstream open reading frame encoding a putative 172-aa protein is flanked by 2 tandem copies of IS*26*. This arrangement is identical to that in pXZ and pHN7A8, except that pXZ carries *bla*_CTX-M-24_ instead of *bla*_CTX-M-65_. The *bla*_CTX-M-24_ and *bla*_CTX-M-65_ gene products differ by only 1 aa (Val for CTX-M-65 and Ala for CTX-M-24 at position 77).

A total of 6 *E. coli* isolates were collected from the patient during 2007–2010, including the isolates reported here and previously (*4*). All isolates were obtained from urine samples, except for YD472, which was cultured from the peritoneal catheter exit site. Pulsed-field gel electrophoresis (PFGE) with *Xba*I as the restriction enzyme showed that all isolates had identical banding patterns. Furthermore, 5 of the 6 isolates, including the first isolate from 2007, were resistant to fosfomycin and positive for *fosA3*, *rmtB*, and *bla*_CTX-M-9-group_ by PCR. *E. coli* TOP10 transformants harboring *fosA3*-carrying plasmids were generated from each of these 5 isolates.

**Figure 2:**
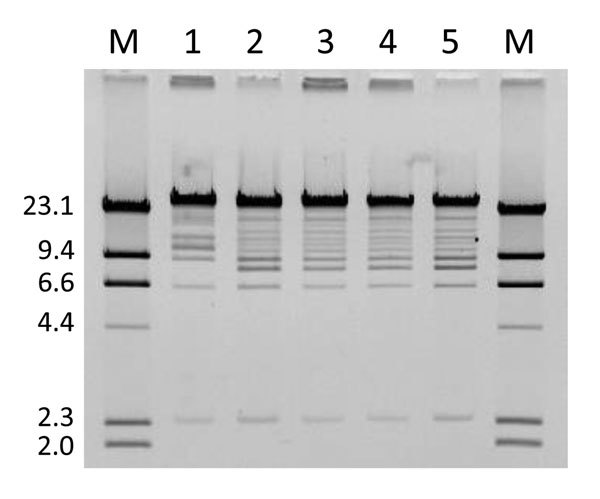
*Eco*RI restriction profile of *fosA3*-carrying plasmids from a woman in Pennsylvania, USA, who was colonized with fosfomycin-resistant *Escherichia coli*. Lanes M, lambda DNA/*Hin*dIII marker; lane 1, February 2011 (YD472); lane 2, March 2008; lane 3, June 2007 (ECRB1); lane 4, April 2008; lane 5, January 2008. Values on the left are in kilobases.

The plasmids were then extracted and digested with restriction enzyme *Eco*RI. The resulting restriction profile was nearly identical across the plasmids except for pYHCC from YD472, which had a shift of a band from ≈8 kb to ≈11 kb (Figure 2). This shift approximated the size difference between pYHCC and pXZ, and further suggested pXZ as the origin of pYHCC.

## Conclusions

The first reported clinical *E. coli* isolate carrying *fosA3* was identified in Japan in 2006 (*3*), but *fosA3*-carrying *E. coli* isolates have also been identified in pigs in China as far back as in 2004 (*14*). Although the patient in Pennsylvania lacked a relevant travel history, the timeline and structural similarities of these *fosA3*-carrying plasmids make it plausible that this multidrug resistance plasmid originated in *E. coli* in Asia and moved to the continental United States by human travel or importation of food products. Emergence of *E. coli* with high-level fosfomycin resistance is a clinically relevant event, especially in the United States, where fosfomycin is increasingly used for empiric treatment of urinary tract infection in the absence of routine drug susceptibility testing.
